# A Review on the Application of Biosensors for Monitoring Emerging Contaminants in the Water Environment

**DOI:** 10.3390/s25164945

**Published:** 2025-08-10

**Authors:** Yi Xiao, Zhe Du, Yuqian Li, Lijia Cao, Bo Zhu, Tetsuya Kitaguchi, Caihong Huang

**Affiliations:** 1 State Key Laboratory of Environmental Criteria and Risk Assessment, Chinese Research Academy of Environmental Sciences, Beijing 100012, China; 2 State Environmental Protection Key Laboratory of Ecological Effect and Risk Assessment of Chemicals, Chinese Research Academy of Environmental Sciences, Beijing 100012, China; 3 College of Safety and Environmental Engineering, Shandong University of Science and Technology, Qingdao 266590, China; 4 Institute of Yellow River Delta Earth Surface Processes and Ecological Integrity, Shandong University of Science and Technology, Qingdao 266590, China; 5 Laboratory for Chemistry and Life Science, Institute of Integrated Research, Institute of Science Tokyo, 4259 Nagatsuta-cho, Midori-ku, Yokohama 226-8501, Kanagawa, Japan

**Keywords:** biosensors, emerging contaminants, environment monitoring, sensitivity, detection limit, water safety

## Abstract

Due to the frequent occurrence and elevated concentrations of emerging contaminants (ECs) in water environments, as well as their high toxicity, these compounds have become a growing concern, threatening water safety, human health, and environmental health. Stricter regulations and routine monitoring are required to control EC pollution in water. Analytical chemistry-based techniques are the most widely used approach for quantifying ECs in environmental samples. However, high costs, complex sample preparation, time-consuming protocols, and labor-intensive processes limit their application for the routine and rapid detection of ECs. Biosensors are a promising biotechnological alternative that has received increased attention in recent years for the quantification of ECs. This review provides a comprehensive overview of the main types of biosensors used for monitoring ECs in aquatic environments, highlighting their underlying detection mechanisms and recent technological advancements. It also discusses key challenges associated with different biosensor platforms, such as stability, sensitivity, and development complexity. Potential future research directions to address these limitations and enhance the performance of biosensors include immobilization on hybrid nanomaterials, and the development of portable and multifunctional biosensors for on-site and real-time monitoring. By summarizing current progress and identifying future directions, this review will broaden the awareness and recognition of biosensors for monitoring ECs in water environments, contributing to water safety, sanitation, and sustainability.

## 1. Introduction

Access to safe and affordable water is one of the most basic needs for all humans and other beings on our planet. Despite progress, water stress and water scarcity remain significant challenges facing humanity in the 21st century due to climate change and population growth [1]. Water pollution has exacerbated the threat to global water safety, causing various diseases such as diarrhea, cholera, dysentery, typhoid, and polio [2]. In 2022, at least 2.2 billion people experienced different types of contamination in their drinking water [3]. Therefore, the sixth Sustainable Development Goal, among the seventeen set by the United Nations, is to ensure access to water and sanitation for all [4]. In addition to conventional pollutants, over the past decade, there has been a growing concern that emerging contaminants (ECs) may pose an even greater threat to water safety, human health, and environmental health. ECs consist of synthetic or natural chemical compounds originating from anthropogenic activities such as industrial, domestic, healthcare, agricultural, and wastewater treatment processes [5], which are not routinely monitored or regulated. Common ECs detected in the water environment include compounds found in pesticides, antibiotics, endocrine disruptors (EDCs), persistent organic pollutants (POPs), and per-and polyfluoroalkyl substances (PFAS) [6,7]. The detection range of the above-mentioned biosensors for pesticides, antibiotics, and other ECs ranges from ng/L to g/L. However, even at concentrations as low as ng/L, ECs can adversely affect human health and other beings, causing issues such as endocrine disruption, mutagenesis, carcinogenesis, and congenital disorders [8,9]. Thus, fast and accurate detection for the control and regulation of ECs contamination is an urgent need for water safety, sanitation, and sustainability.

Analytical chemistry-based techniques are the most widely used approaches for quantifying ECs in water, soil, and other environmental samples [10]. These include high-performance liquid chromatography (HPLC), gas chromatography (GC), mass spectrometry (MS), inductively coupled plasma mass spectrometry (ICP-MS), and atomic absorption spectroscopy (AAS) [11,12]. These methods are generally considered to provide accurate, sensitive, and reliable results. However, their extensive use for routine daily measurements is limited by several factors, such as the high cost of required instruments, complex sample preparation, time-consuming protocols, and labor-intensive processes [13,14,15,16]. These limitations hinder real-time or prompt in situ monitoring of ECs in the water environment, thereby delaying decision-making and potentially leading to greater contamination problems.

**Figure 1:**
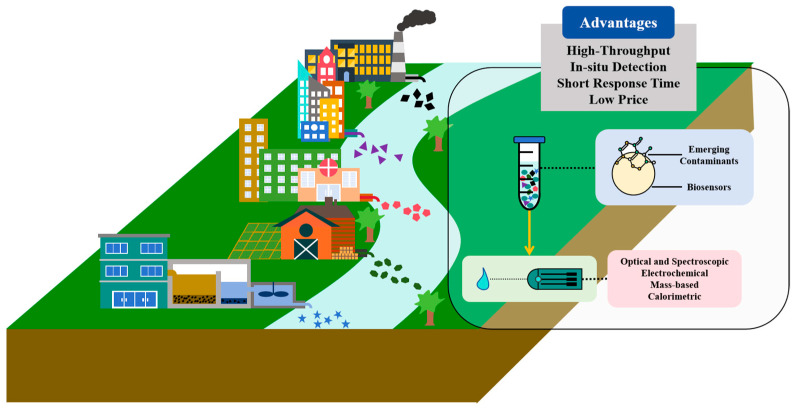
Application of biosensors for monitoring pollutants from various sources (industrial, domestic, healthcare, agricultural, and wastewater treatment plants) in the water environment, with an overview of detection processes and advantages.

To address these challenges, beyond the advances in conventional technologies, many innovative bio-based technologies have been developed for measuring ECs in the water environment. Among these, biosensors represent a promising approach, offering numerous advantages, such as low cost, simplicity, fast processing, sensitivity, and portability (Figure 1) [17,18,19,20]. Depending on the types of functional sensing elements (bioreceptors) used, biosensors can be categorized as enzyme-based, antibody-based, nucleic acid-based, and whole cell-based. Biosensors have been successfully applied in the detection of heavy metals, microorganisms, antibiotics, pesticides, and other contaminants in the water environment [21,22,23,24]. Furthermore, real-time monitoring of drug/pharmaceutical levels in sewage has been realized by combining biosensors and wastewater epidemiology [25]. Extensive studies are exploring the potential of this technique for various types of contaminants in the water environment.

In this review, we aim to provide a comprehensive overview of recent advancements in the application of biosensors for pollution monitoring in the water environment, with a particular focus on ECs. Initially, we offer a brief summary of different types of biosensors and their corresponding working mechanisms. Subsequently, we review and discuss the recent progress in using biosensors for monitoring ECs in the water environment. Finally, we discussed the current challenges and prospects of biosensors in ECs assessment. The review aims to support ongoing research and potential practical applications in the field of biosensor-based monitoring of ECs, highlighting both analytical progress and the remaining challenges.

## 2. Types of Biosensors and Working Mechanisms

### 2.1. Enzyme-Based Biosensors

**Figure 2:**
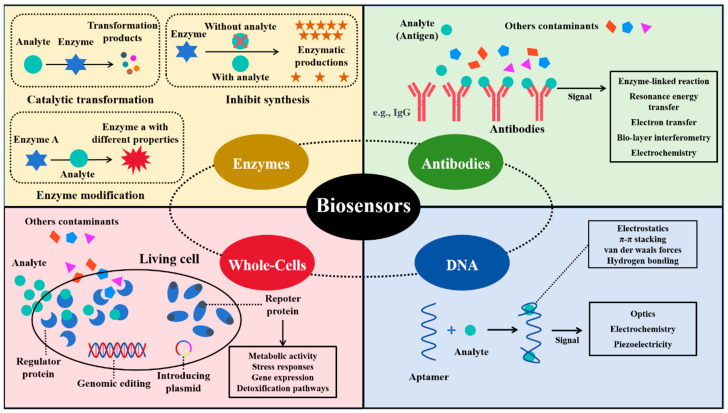
Types and working mechanisms of biosensors based on bioreceptors.

Enzyme-based biosensors employ enzymes as bioreceptors to catalyze reactions with the target analyte, producing a detectable signal [26]. The binding capabilities of the analyte are crucial for the performance of these biosensors [27]. There are several different biorecognition mechanisms: (1) the enzyme metabolizes the analyte, allowing the analyte concentration to be estimated by its catalytic transformation; (2) the enzyme is inhibited by the analyte, so the analyte concentration is correlated with the reduction in enzyme product synthesis; and (3) the analyte affects certain characteristics of an enzyme, and such change can be used for quantification assessment [28,29,30,31] (Figure 2). As one type of the earliest developed biosensors, enzyme-based biosensors exhibit high specificity and sensitivity for target compound detection in different applications. The biocatalytic reaction in enzyme-based biosensors typically produces electrical, optical, or thermal signals for the quantification of analytes. Among these, electrochemical transducers are the most commonly used, due to their advantages of being rapid, simple, and portable [32].

### 2.2. Antibody-Based Biosensors

Antibody-based biosensors, often referred to as immunosensors, utilize the high specificity and affinity of antibodies for target recognition, making them ideal biorecognition elements in biosensor platforms. Antibodies are highly soluble serum glycoproteins also known as immunoglobulins (Ig). Based on the type of heavy chain in Ig, they can be categorized as IgG, IgM, IgA, IgD, and IgE [33]. Antibody-based biosensors can be categorized into label-free and labeled systems depending on their signal transduction mechanisms [34] (Figure 2). Label-free biosensors detect physical changes introduced by the antigen–antibody binding events, such as changes in impedance, refractive index, or mass. For example, Ionescu et al. developed an impedimetric immunosensor to detect ciprofloxacin (CIP) antibiotics, in which the formation of antigen–antibody complex on an electrode surface can directly trigger a measurable impedance spectroscopic signal, achieving a detection limit as low as 10 pg/mL [35]. In contrast, labeled biosensors utilize secondary molecules to generate detectable signals upon binding, such as fluorescence dyes, enzymes, or nanoparticles. For instance, Song et al. developed a biosensor using a multicolor quantum dot (QD) fluorescence immunoassay analysis technique for determining multiple antibiotic residues in milk [36]. In this competitive immunoassay, unbound antibodies bind to QD probes, forming a QD-Ab complex that yields fluorescence signals for quantification.

### 2.3. Nucleic Acid-Based Biosensors

Aptasensors are a type of nucleic acid-based biosensor that utilize synthetic single-stranded DNA or RNA aptamers as recognition elements to bind specific target analytes. These biosensors rely on the high affinity and specificity of aptamers, which are selected through SELEX, to enable the selective detection of various environmental contaminants. [37]. The aptamer–analyte complex can fold into two-dimensional (2D) or three-dimensional (3D) structures, enhancing the stability and performance of the biosensors due to higher surface density and reduced spatial blocking [38,39,40]. Aptasensors bind to their targets through various electrostatic and intramolecular mechanisms, including π-π stacking, van der Waals forces, and hydrogen bonding, allowing them to recognize a wide variety of analytes, such as metal ions, proteins, organic compounds, and even living cells or tissues, thereby triggering signal transformation (Figure 2). Signal transduction in aptasensors usually relies on optical, electrochemical, and piezoelectric techniques. Aptasensors can be synthesized through chemical processes, which are generally considered simpler than the biological synthesis systems used to produce enzyme-based or antibody-based biosensors. Aptamers are usually isolated from oligonucleotide libraries through in vitro selection mechanisms such as Systematic Evolution of Ligands by Exponential Enrichment (SELEX) [41,42].

### 2.4. Whole Cell-Based Biosensors

The biorecognition elements of whole cell-based biosensors are microbial cells, such as bacteria, fungi, algae, and protozoa. These cells function as integrated machinery, possessing both receptors and transducers [43]. One of the unique features of cell-based biosensors is their ability to self-replicate, producing more biorecognition elements which can enhance signal detection over time [44,45]. Compared to other types of biosensors, cell-based biosensors are typically more robust across various application conditions and are easier to handle. Microbial cells can be easily engineered through genomic editing or by introducing a plasmid to tailor the sensing system to meet the requirements of different types of analytes [46]. Thus, various working mechanisms can be found in cell-based biosensors, including those based on metabolic activity, stress responses, gene expression regulation, and detoxification pathways (Figure 2). Benefiting from the advantages of cell-based biosensors, they have been successfully applied in fields such as environmental monitoring, food analysis, pharmacology, drug screening, and the detection of heavy metals, pesticides, and organic contaminants [47]. For example, Riangrungroj et al. used *Escherichia coli* (*E. coli*) to build a label-free cell-based biosensor with an optical transducer for monitoring pyrethroid insecticide, achieving a detection limit of 3 ng/mL [48].

## 3. Application of Biosensors for Monitoring ECs in Water Environments

### 3.1. Pesticides Monitoring

Pesticides are chemical substances commonly used in agriculture to control pests, diseases, and weeds that may threaten crop production. These chemicals include herbicides, insecticides, fungicides, and rodenticides, which are vital for maintaining high agricultural yields and food security [49,50]. Misuse or overuse of these chemicals is fairly common, and they may eventually enter the environment through runoff, drift, or leaching, causing potential water contamination and certain human health impacts. Organochlorines, organophosphates, carbamates, pyrethroids, and neonicotinoids are the most commonly detected compounds in surface water and groundwater worldwide [51,52]. Table 1 summarizes the applications of biosensors based on four different bioreceptors in the detection of pesticides. Among these compounds, organochlorines are usually considered to have greater toxicity, due to their high solubility in water and persistence in the environment. Previous study suggests that they have certain carcinogenic and mutagenic effects [53].

#### 3.1.1. Enzyme Biosensors for Pesticide Monitoring

Enzyme-based biosensors have been widely applied for detecting various pesticide compounds in aquatic environments. These sensors leverage the fact that many pesticides exert their toxicity by inhibiting key enzymes involved in biological processes, a mechanism that can be directly harnessed for detection. Specific enzymes have been extensively employed due to their well-characterized and selective interactions with different pesticide classes. For instance, acetylcholinesterase (AChE) is commonly used to detect organophosphorus pesticides through inhibition-based sensing. Other enzymes, such as butyrylcholinesterase (BChE), organophosphorus hydrolase (OPH), tyrosinase, laccase, peroxidase, alkaline phosphatase (ALP), and urease, are selected based on their catalytic compatibility with the chemical structures or functional groups of various pesticides [76,77,78]. These biosensors typically rely on electrochemical, optical, or piezoelectric transduction methods to convert enzymatic activity changes into quantifiable signals [79,80]. Among these, electrochemical approaches dominate due to their compatibility with portable formats and relatively simple fabrication [81].

While enzyme-based biosensors offer simplicity and affordability, their broader application is constrained by issues such as limited operational stability, sensitivity to environmental conditions, and narrow substrate specificity. Enzyme activity can be affected by pH, temperature, and matrix interferences, potentially reducing accuracy and reproducibility in complex environmental samples. Additionally, traditional single-enzyme sensors may struggle to distinguish among structurally similar pesticide compounds, limiting their versatility in multi-residue analysis.

In response to these challenges, recent studies have aimed to improve enzyme robustness and expand detection capabilities through stabilization strategies and multi-enzyme designs. For example, Ma et al. encapsulated aryloxyphenoxypropionate esterase and alcohol oxidase in Fe_3_O_4_-modified ZIF-8 nanocrystals to detect multiple herbicides [82]. Enzyme immobilization has also emerged as a key strategy to enhance biosensor stability and reusability. By embedding enzymes within polymer matrices or nanomaterials, such as gelatin, graphene, or gold nanoparticles, researchers have achieved improved resistance to degradation, extended operational lifetimes, and sub-nanomolar detection limits [83,84,85]. Although promising, most enzyme-based biosensors have been validated under controlled conditions, and further efforts are needed to ensure their reliable performance in field-relevant, complex environmental samples.

#### 3.1.2. Immunosensors for Pesticide Monitoring

Owing to the high-affinity binding between antigens and antibodies, immunosensors are widely regarded as exhibiting the highest specificity among various biosensor types, enabling the sensitive and selective identification of target analytes. Pesticide compounds are typically considered haptens, as they are incapable of eliciting an immune response independently. When conjugated to larger carrier molecules, such as bovine serum albumin (BSA) proteins, these compounds acquire immunogenicity [86]. BSA is frequently used due to its high stability, low cost, abundant amino groups, and solubility in organic solvents [87]. In immunosensors, competitive immunoassays are preferred for small molecules such as pesticides, which typically have a single epitope, whereas sandwich assays are more suitable for larger targets. For example, a competitive fluorescence immunosensor employing catalytic hairpin assembly (CHA) amplification was developed for detecting triazophos, achieving a detection range of 0.01–50 ng/mL and a limit of detection (LOD) of 0.03 ng/mL [88].

Despite their promising sensitivity, immunosensors face distinct challenges beyond general biomolecule instability. These include the high cost, time-consuming nature of antibody production, and limited reusability. Furthermore, environmental factors such as temperature, pH, and organic matrix components can adversely affect antibody binding efficiency and sensor performance.

**Figure 3:**
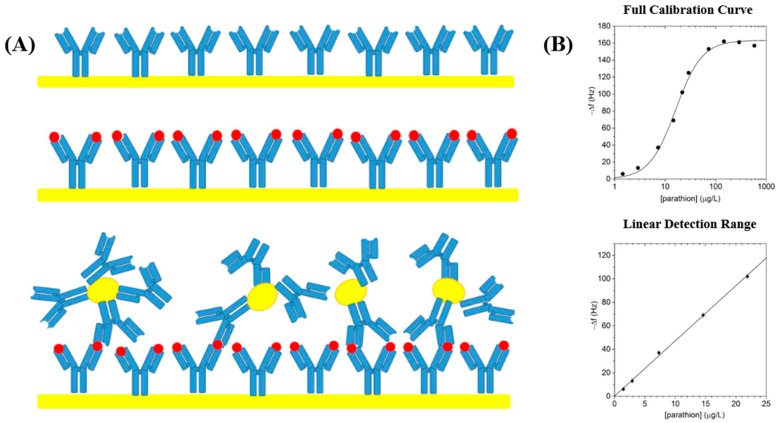
Immobilization of immunosensors on a solid surface combined with nanoparticles developed by Ventura et al. for the detection of pesticides. (**A**) Schematic representation of antibody immobilization on a quartz crystal microbalance surface with gold nanoparticles for parathion detection. (**B**) The top plot shows the full calibration curve of the biosensor response to parathion over a wide concentration range (0–1000 μg/L) in logarithmic scale; the bottom plot highlights the linear detection range of the sensor, demonstrating a strong linear correlation in the lower concentration range of 0–25 μg/L, suitable for quantitative analysis. Copyright PLoS One, 2017 [89].

To address these limitations, recent studies have also utilized nanomaterial integration to improve antibody stability, extend shelf life, and enhance signal transduction. Among them, carbon-based nanomaterials have attracted particular attention for their dual functionality, such as metal nanoparticles, carbon dots, graphene QDs, and g-C_3_N_4_ nanosheets. They not only help stabilize antibodies but also serve as effective fluorescent signal amplifiers, significantly enhancing sensor sensitivity. For example, one study immobilized the primary antibody on the surface of a quartz crystal microbalance (QCM) and conjugated the second antibody with gold nanoparticles to amplify the signal, enabling the analysis of small-molecule compounds (Figure 3A). This biosensor was tested with parathion concentrations ranging from 0 to 10,000 μg/L, and a stable and well-defined linear response was observed at low concentrations (0–25 μg/L) (Figure 3B). This biosensor demonstrates high specificity, showing low cross-reactivity with parathion analogs [89]. Similarly, the development of monoclonal antibodies has become a preferred approach to further improve target specificity and reduce cross-reactivity [90]. Additionally, the integration of immunosensors with microfluidic platforms has enabled more rapid, on-site detection, advancing their potential for field applications [91].

#### 3.1.3. Aptasensors for Pesticide Monitoring

Aptasensors utilize short single-stranded DNA or RNA sequences, typically 15 to 100 nucleotides in length, with dissociation constants ranging from micromolar to picomolar, depending on the targets [92]. By binding pesticides with high affinity and specificity, these sequences undergo conformational changes that are transduced into measurable signals via electrochemical, optical, or mass-sensitive platforms. For pesticide detection, fluorescence or electrochemical methods are most commonly employed, particularly for small molecules such as organophosphates [93]. Unlike immunosensors, aptasensors can bind directly to small pesticide molecules without the need for conjugation to carrier proteins. Well-designed aptamers often surpass antibodies in binding affinity and specificity [92], while offering key advantages such as greater thermal and chemical stability, cost-effective chemical synthesis, and easier modification [94]. Recognition mechanisms include direct target binding (formation of aptamer-analyte complexes) and competitive binding (analyte displacing a labeled probe). For instance, an indirect competitive aptamer-based enzyme-linked immunosorbent assay (apt-ELISA) was developed for detecting isocarbophos residues in environmental samples. In this system, isocarbophos competes with a labeled complementary strand for aptamer binding, resulting in a measurable signal change [95]. Similarly, some studies have prepared a competitive probe composed of magnetic Fe_3_O_4_ nanoparticles and complementary chain DNA (cDNA) for the quantitative detection of carbendazim [96].

Although aptasensors offer notable advantages in selectivity, stability, and adaptability, several technical limitations continue to hinder their broader application. The SELEX process is labor-intensive and must be tailored individually for each target, making it difficult to scale up across diverse pesticide classes. Moreover, aptamers that perform well in buffer or spiked solutions often suffer reduced structural stability and binding efficiency in complex environmental matrices such as wastewater or sediments, where nonspecific interactions and chemical interference can compromise the performance.

To enhance robustness and analytical performance, researchers have increasingly turned to nanomaterial-assisted designs. Materials such as gold nanoparticles (AuNPs), single-walled carbon nanotubes (SWNTs), and streptavidin-coated silica nanoparticles (SiNPs) have been used to amplify detection signals, improve aptamer immobilization, and increase sensitivity [97,98,99,100]. These technological advancements, along with aptamers’ inherent modifiability and compatibility with portable platforms, continue to position aptasensors as a promising tool for pesticide monitoring in aquatic environments.

#### 3.1.4. Whole-Cell Biosensors for Pesticide Monitoring

Cell-based biosensors utilize living cells as biorecognition elements to detect pesticides based on biological response changes in metabolism, viability, or gene expression, with commonly used hosts, including bacteria [71] and yeast [101]. A key advantage of these biosensors is their ability to capture holistic microbial physiological responses, such as metabolic disruption or stress induction, offering a systems-level view of toxic effects. In addition, the presence of intact cellular membranes and regulatory systems confers inherent robustness, helping buffer against matrix interferences and environmental fluctuations [102]. These systems are also cost-effective, as genetically engineered cell lines can be readily propagated and maintained with relatively low resource input under standard laboratory conditions [103]. When pesticides interact with cells, the induced biological changes can be transduced into measurable electrochemical, optical, or fluorescence signals. For instance, Teng et al. developed a biosensor using a fusion protein of OPH and pHluorin (a pH-sensitive green fluorescent protein) [104]. Upon hydrolysis of organophosphorus pesticides by OPH, the resulting pH alters the fluorescence intensity of pHluorin, allowing for sensitive detection.

Although cell-based biosensors offer unique advantages, their broader implementation in environmental pesticide monitoring is constrained by several technical and practical challenges. While cells have intrinsic buffering mechanisms that offer a degree of robustness, maintaining cell viability and ensuring consistent response under variable field conditions can sometimes be problematic. Additionally, the biological nature of the sensing element may lead to batch-to-batch variability and slower response times compared to molecular sensors.

To improve sensor stability and operational consistency, various immobilization strategies have been employed, including embedding microbial cells in sol-gel matrices or encapsulating them within diatom-derived silica shells. These approaches provide physical protection while preserving permeability and biological function, supporting reuse and long-term monitoring [105]. However, their regeneration and operational lifetime still need further optimization for reliable field applications. Microfluidic integration has emerged as a hot research area, enhancing the throughput and multiplexing capacity of cell-based biosensors [106,107]. Additionally, synthetic biology tools now allow the engineering of cells with pesticide-specific genetic circuits (e.g., inducible promoters or reporter systems), further improving specificity and sensitivity. This genetic tunability is a major advantage for designing customizable and responsive biosensing platforms suited to complex environmental conditions.

### 3.2. Antibiotics Monitoring

Since the discovery of antibiotics in the last century, their usage has increased significantly worldwide, saving countless lives. However, antibiotics are usually partially digested or absorbed by humans and animals. A large portion of them, ranging from 10% to 90%, is excreted in urine or feces and eventually enters different environmental media such as water, soil, sediments, and air [108]. The occurrence of antibiotics in surface water has been continuously reported over the past two decades. A global dataset was recently established to record antibiotic contamination in surface water. According to this dataset, 169 types of antibiotics have been detected in water environments across 63 countries on different continents, including all major antibiotic categories: sulfonamides, tetracyclines, quinolones, β-lactams, and macrolides. Among them, sulfamethzine (SMZ), sulfamethoxazole (SMX), CIP, norfloxacin (NOR), and erythromycin (ETM) are the most frequently detected due to their extensive usage, with the concentration up to 10–100 µg/L [109]. Antibiotics can create selective pressure on microbial communities and ultimately alter the natural balance of microbial community composition by enriching microorganisms containing certain antibiotic resistances, such as those promoting harmful algal blooms. Furthermore, they can stimulate the antibiotic gene transfer through different mechanisms, leading to the production of “superbugs” [110,111,112,113]. Therefore, strict regulation of antibiotic use and close monitoring of the health risk posed by antibiotics are necessary to protect all beings and the environment on our planet. In recent years, biosensors have emerged as a promising approach for monitoring antibiotics in water environments in a fast and cost-effective manner. Table 2 summarizes the detection limits, response times, and detection ranges of different types of biosensors for detecting antibiotics.

#### 3.2.1. Enzyme Biosensors for Antibiotic Monitoring

Enzyme-based biosensors for antibiotic detection typically employ biocatalysts such as penicillinase (β-lactamase) [135], glucose oxidase (GOx) [136], horseradish peroxidase (HRP) [137], and tyrosinase [138] to catalyze antibiotic-related reactions. Amperometric biosensors using penicillinase are widely applied for detecting β-lactam antibiotics, while HRP-based platforms have been extended to other antibiotic classes, including tetracyclines [139], chloramphenicol [140], sulfonamides [141], and aminoglycosides [142].

**Figure 4:**
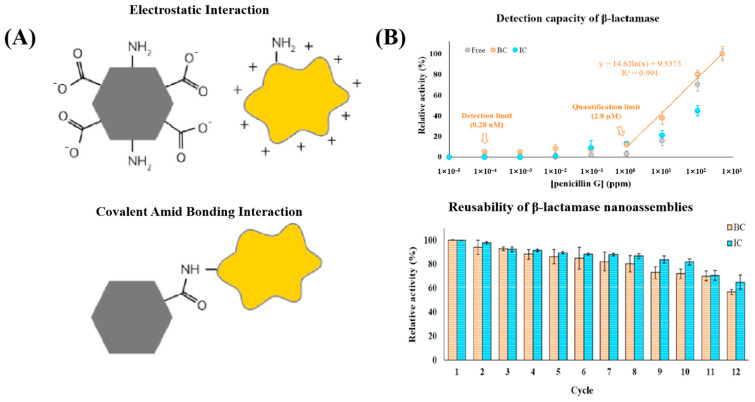
Enzyme-based biosensors combined with biomimetic magnetic nanomaterials developed by Jimenez-Carretero et al. for the detection of antibiotics with potential reusability. (**A**) Schematic representation of the electrostatic and covalent amid bonding interaction mechanisms between β-lactamase (orange polygons) and biomimetic magnetic nanomaterials (black regular hexagons). (**B**) Detection performance and reusability of β-lactamase nanoassembled biosensors for penicillin G quantification. The enzyme β-lactamase was covalently immobilized onto biofunctionalized magnetic nanoparticles (BMNPs) (BC: BMNP-covalently bound) and inorganic magnetic nanoparticles (IC: inorganic MNP-covalently bound). The BC nanoassemblies exhibited a linear response across a penicillin G concentration range of 1 to 500 ppm, with a detection limit of 1 ppm. Reusability tests showed that β-lactamase retained approximately 57 ± 2% of its initial activity after 12 washing cycles. Copyright Reprinted Talanta, 2023 [144].

To enhance sensitivity, extend detection ranges, and improve operational stability, enzyme-nanomaterial hybrids have been developed for antibiotic monitoring. For example, Tang et al. constructed a biosensor using metal β-lactamase to hydrolyze ampicillin’s β-lactam ring, generating electroactive metabolites that modulate local pH and produce detectable electrochemical signals [143]. The enzyme was immobilized in a composite matrix of chitosan, Bi_2_WO_6_, and carboxylated multi-walled carbon nanotubes (MWCNTs-COOH). Bi_2_WO_6_ enhanced conductivity and catalytic efficiency, while MWCNTs increased surface area and electron transfer, resulting in a LOD of 0.05 μM. Similarly, magnetic nanoparticles (MNPs) have gained attention as enzyme carriers due to their magnetic responsiveness and reusability. However, conventional MNPs often require surface modification (e.g., with PEG or organic acids) to maintain enzyme activity, which can impair their magnetic properties. To overcome this, biomimetic magnetic nanoparticles (BMNPs) mediated by proteins such as MamC have been developed, offering superparamagnetism, optimal sizes (30–40 nm), and high magnetic moments. Jimenez-Carretero et al. demonstrated the use of BMNPs functionalized with β-lactamase to detect penicillin G via UV-visible absorbance changes, achieving a LOD of 1 ppm and a linear range of 1–500 ppm. β-lactamase covalently bound to BMNPs retained 57 ± 2% of its initial activity after 12 repeated uses (Figure 4B) [144]. Additional improvements have also been achieved through integration with microfluidic systems for rapid analysis and machine learning tools to enhance data processing and specificity [145,146]. Despite these promising advances, enzyme-based biosensors for antibiotics still face challenges in field applications due to matrix complexity and fluctuating environmental conditions.

#### 3.2.2. Immunosensors for Antibiotic Monitoring

Immunosensors exploit the selective interaction between antibodies (or antibody fragments) and antibiotic targets such as β-lactams, tetracyclines, chloramphenicol, sulfonamides, aminoglycosides, and macrolides, producing electrochemical or optical signals upon binding. Electrochemical immunosensors detect surface property changes using techniques such as cyclic voltammetry (CV), differential pulse voltammetry (DPV), and electrochemical impedance spectroscopy (EIS), such as current, voltage, or impedance [147,148]. In contrast, surface plasmon resonance (SPR) enables real-time, label-free detection by monitoring changes in the refractive index during binding events [149].

The integration of nanomaterials such as carbon nanotubes, graphene, and gold nanoparticles into immunosensor platforms has been widely developed to enhance signal amplification and lower detection limits. For instance, Zhang et al. developed a highly sensitive electrochemical immunosensor for sulfonamides using gold nanodendrites (AuNDs) as a sensing platform and AgNPs@single-walled carbon nanohorns (AgNPs@SWCNHs) as signal probes [119]. This indirect competitive assay used coated antigens, antibodies (Ab1), and secondary AgNP-conjugated antibodies (Ab2) to generate silver ion release upon nitric acid addition, yielding a LOD of 0.12 ng/mL for SMZ with demonstrated effectiveness in real water samples. More recent developments include QD-based fluorescence immunoassays [150,151], the integration of microfluidics for rapid point-of-use detection [152], and machine learning-based signal analysis for enhanced specificity in complex matrices [153].

#### 3.2.3. Aptasensors for Antibiotic Monitoring

Aptasensors are versatile in binding both small-and large-molecule antibiotics, and have been widely developed to detect a range of targets such as β-lactams, aminoglycosides, anthracyclines, chloramphenicol, quinolones, tetracyclines, and sulfonamides [154]. After binding to antibiotics, aptamers undergo conformational changes that are transduced into measurable signals via electrochemical, optical, or mass-sensitive platforms [154,155,156]. Compared to conventional electrochemical sensors, photoelectrochemical (PEC) aptasensors incorporating QDs offer improved signal stability and tunability, owing to the unique optical and electronic properties of QDs [157,158]. For example, a PEC aptasensor utilizing graphene-doped BiOI (BiOI-G) as the photoactive material demonstrated a detection limit of 0.9 nM and a linear range of 4.0–150 nM for oxytetracycline (OTC) [159]. A fluorescent aptamer sensor was developed using graphene oxide (GO) and cadmium selenide quantum dots (CdSe QDs). In this system, GO binds to the aptamer labeled with CdSe QDs, resulting in fluorescence quenching. When ciprofloxacin in the sample specifically binds to the aptamer, the quencher is displaced, and fluorescence is restored, enabling the quantitative detection of ciprofloxacin. The detection limit of this aptamer sensor is 0.42 nM [160].

For antibiotic detection using aptasensors, signal enhancement techniques have been developed to significantly improve detection sensitivity for trace-level targets. These include strand displacement reactions, rolling circle amplification (RCA), and hybridization chain reactions (HCR) [161,162,163]. In parallel, multiplexed aptasensors have been designed to simultaneously detect multiple antibiotic residues in environmental samples. For instance, a FRET-based aptasensor combining DNAzymes with catalytic strand displacement amplification (SDA) enabled the ultrasensitive detection of tetracycline, chloramphenicol, and kanamycin in water samples [161]. Ongoing research also focuses on the chemical modification of aptamers to enhance structural stability, as well as integration with artificial intelligence for predictive signal interpretation and adaptive biosensor design [164,165,166].

#### 3.2.4. Whole-Cell Biosensors for Antibiotic Monitoring

**Figure 5:**
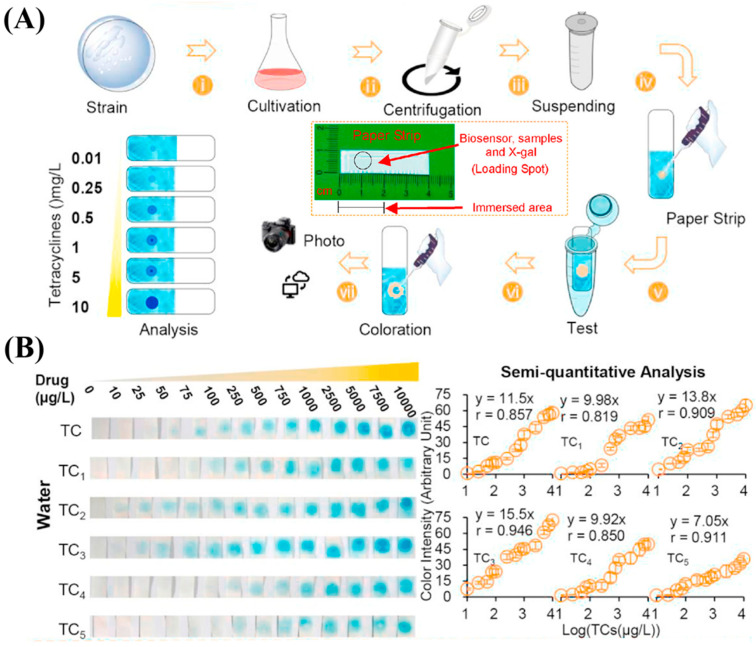
Portable Whatman filter paper strip biosensors developed by Ma et al. for tetracycline antibiotic detection using engineered *E. coli* whole-cell biosensors. (**A**) The detection process involves culturing *E. coli* harboring the pMTLacZ plasmid, spotting the cells onto filter paper, freeze-drying, rehydrating in antibiotic-containing LB medium, and adding X-gal. In the presence of tetracyclines, the repression by *TetR* is relieved, allowing expression of the *lacZ* gene and production of β-galactosidase, which hydrolyzes X-gal into a blue-colored compound (5,5′-dibromo-4,4′-dichloro-indigo). (**B**) curves for six tetracycline-type antibiotics (TC–TC_5_), showing detection limits (μg/L) and linear response ranges (μg/L) as follows: TC (5.86; 25–10,000), TC_1_ (5.85; 75–10,000), TC_2_ (5.44; 25–10,000), TC_3_ (5.23; 10–10,000), TC_4_ (17.1; 75–10,000), and TC_5_ (9.05; 75–10,000). Copyright Biosensors and Bioelectronics, 2020 [132].

Living cells, such as *E. coli* or genetically engineered strains, have been used to detect antibiotics through biologically mediated responses. These biosensors respond to antibiotic-induced cellular disruptions, which can be transduced into measurable signals via electrochemical (e.g., impedance), optical (e.g., luminescence), or fluorescence-based methods, such as inhibition of protein synthesis or induction of stress pathways. Among these, paper-based whole-cell biosensors have gained attention due to their simplicity, portability, and disposability. For instance, a paper sensor constructed on Whatman filter paper incorporated *E. coli*/pMTLacZ containing a tetracycline-regulated gene and β-galactosidase as a reporter. Upon exposure to tetracycline, the antibiotic binds to the TetR repressor, triggering the expression of the lacZ gene. The resulting β-galactosidase catalyzes the hydrolysis of X-gal, producing a blue color that enables semi-quantitative visualization of tetracyclines in environmental water samples. After 90 min of incubation, the biosensor exhibited linear response ranges for six tetracycline compounds, including tetracycline (25–10,000 μg/L), oxytetracycline (75–10,000 μg/L), chlortetracycline (25–10,000 μg/L), deoxytetracycline (10–10,000 μg/L), minocycline (75–10,000 μg/L), and methicillin (75–10,000 μg/L) (Figure 5B) [132]. Also targeting cellular gene expression, Liu et al. used synthetic biology technology to study a type of engineered paper cell biosensor. After improving gene expression, they achieved the quantitative analysis of tetracycline in tap water (5–1280 μg/mL) [167].

Building on synthetic biology tools, recent work has focused on engineering antibiotic-specific genetic circuits and developing alternative sensing platforms to improve specificity and operational robustness. For example, a whole-cell biosensor incorporating a superfolding green fluorescent protein (sfGFP) reporter and optimized T7-tetO promoter spacing achieved a detection limit of 0.0097 mg/L for tetracycline [168]. Beyond whole-cell systems, cell-free expression (CFE) platforms have emerged as promising alternatives by eliminating the physiological complexity of intact cells while retaining programmable gene expression. A riboswitch-based CFE fluorescence biosensor was developed for tetracycline detection, achieving a response time of 80 min and retaining functionality for 15 days at room temperature after lyophilization and rehydration [169]. These features broaden the potential for field deployment and point-of-use diagnostics. Ongoing research in CFE systems aims to improve stability, expand the range of detectable targets, and create user-friendly, portable sensor formats [170].

### 3.3. Other Pollutants

The exploration and application of biosensors for detecting other ECs compounds in EDCs, POPs, and PFAS in water environments is not as extensive as for pesticides and antibiotics. Table 3 summarizes the latest progress in the use of biosensors for monitoring EDCs, POPs, and PFAS.

#### 3.3.1. EDCs

EDCs are a class of compounds that affect the normal operation of the endocrine system in organisms by imitating natural hormones or inhibiting the binding of hormones to receptors, causing a series of diseases, including diabetes, infertility, obesity, neurodegenerative diseases, heart disease, and thyroid dysfunction [189]. Similar to antibiotics, EDCs can easily enter the water environment due to limited digestion and degradation in organisms [190]. EDCs can enter the human body through direct contact, or through the food chain due to bioaccumulation [191,192,193]. Considering the widespread presence of EDCs in environmental media and potential health risk for humans and other beings, EDCs are considered ECs in the US, the European Union, and other nations [194,195,196].

Among EDCs, biosensors have been developed to detect bisphenol A (BPA) in water samples. BPA is one of the most abundant chemicals produced globally as a starting material and plasticizer for polymer production [197]. In 2013, BPA production was around 7 million metric tons and was estimated to increase to more than 9.6 million metric tons by 2020 [198]. For enzyme biosensors, enzyme immobilization is critical. This step requires that the enzyme be closely connected to the carrier and that the enzyme has sufficient activity to react. Chitosan, as a natural, non-toxic, hydrophilic polysaccharide material, can provide a certain strength and has good bioadaptability, but on the contrary, chitosan itself has certain insulating properties, which limits the electronic transfer process of the sensor, thereby affecting the efficiency and results of the detection. Therefore, material modification has become the focus of research. Multiple types of biosensors have been developed for the detection of BPA as an alternative to the commonly used electrochemical approach due to electrode fouling issues [199]. Bravo et al. developed an enzyme-based biosensor for detecting BPA based on the polyphenol oxidase laccase enzyme from Trametes versicolor. A bioconjugate was created using this enzyme (6.8 U/mL), chitosan (5 mg/mL), and the ionic liquid 1-butyl-3-methylimidazolium tetrafluoroborate in a ratio of 5:5:2 (*v*/*v*/*v*). This bioconjugate is immobilized onto a screen-printed carbon electrode (SPCE) modified with MWCNT for BPA detection based on electrochemical signals from the oxidation of BPA. The optimal working pH of the sensor is 5. Under this condition, the accuracy and detection limit can achieve 94.6–97.9% and 8.4 ± 0.3 nM, respectively [200]. Also based on magnetic metal nanomaterials, Hu et al. developed a class of aptamer sensors for BPA detection with a detection limit of 3.3 pg/mL (about 0.0144 nM, based on the molar mass of BPA 228.29 g/mol). These aptamer sensors consist of recognition probes (magnetically reduced graphene oxide with gold nanoparticles, MrGO@AuNPs) and signal probes (gold nanoparticles with methylene blue). BPA competes with the signal probe for binding to the recognition probe, thereby changing the number of electroactive molecules on the electrode and reducing the electron transfer rate, which can be used to quantitatively determine the concentration of BPA [201].

In addition to BPA, 17β-estradiol, testosterone, and androstenedione are common EDCs found in the environment, with concentrations ranging from 0.2 to 3 mg/L in environmental water bodies [202]. Singh et al. developed a capacitive immunosensor that can rapidly detect 17β-estradiol. The sensor is primarily constructed by coupling an electrode with a specific monoclonal antibody (mAb) as the immunological transducer. After 17β-estradiol binds to the mAb, capacitance changes are used for 17β-estradiol quantification. The detection limit can reach 1 pg/mL, with the linear response range of 5–200 pg/mL. The analysis results of the sensor meet the requirements of EU, Codex, and USFDA standards and demonstrate excellent performance [203]. Yildirim et al. developed an aptamer-based optical biosensor for the rapid and sensitive detection of 17β-estradiol in water samples. A known concentration of fluorescence-labeled aptamer was mixed with water samples to bind to the free 17β-estradiol, and then pumped through the optical fiber sensor surface, which has 17β-estradiol immobilized on its surface. The remaining fluorescence-labeled aptamer binds to the optical fiber sensor surface for fluorescence signal detection and quantification of the 17β-estradiol concentration. The sensor has a detection limit of 2.1 nM and exhibits high specificity and reusability [204]. After the quantification of 17β-estradiol, it can be washed off by altering the buffer conditions, allowing for the reuse of aptasensors immobilized on the optical fiber surface.

#### 3.3.2. POPs

POPs are hazardous chemicals that threaten human health and natural ecosystems. Their key characteristics include persistence in the environment, high mobility, bioaccumulation in organisms, and significant health risks to both humans and ecosystems. As a result, many pesticide compounds can be classified as POPs, such as organochlorine pesticides, including DDT, which will not be discussed in this section. Polychlorinated biphenyls (PCBs) are one of the major and well-known POPs, and are highly carcinogenic man-made chemical compounds. They have been widely used in industrial and consumer products due to their excellent flame retardancy, chemical stability, and electrical insulation properties [205]. The production of PCBs was banned in the United States by the Toxic Substances Control Act in 1976 and internationally by the Stockholm Convention on POPs in 2001. However, they continue to be considered contaminants of concern for monitoring and regulation in the environment, due to their long persistence and bioaccumulation effects. Several biosensors have been developed for assessing PCBs in recent years. Alsefri et al. developed a label-free immunosensor based on a self-assembled monolayer-modified electrode for PCB detection using electrochemical signal detection. A PCB–BSA conjugate was used to compete with free PCBs for binding to the anti-PCB polyclonal primary antibody (IgY). A secondary antibody was then used to bind to the PCB–BSA–IgY structure, enhancing the electrochemical signal sensitivity for PCB detection. This sensor has a linear range of 0.011–220 ng/mL and a detection limit of 0.11 ng/mL [206]. The performance of the biosensor can be further improved by nanomaterial modification. In contrast, Chiara et al. [207] used reduced graphene oxide (rGO) and gold nanoparticles (AuNPs) to improve the motor material and prepared a biosensor for detecting low-brominated diphenyl ethers based on the relatively rare glucose oxidase (GOD), realizing the detection of BDE-3, BDE-15, BDE-28, and BDE-47. Similarly, Zeng et al. [208] used gold nanoparticles to immobilize horseradish peroxidase to detect TBBPA-MHEE and TBBPA-DHEE, with detection limits (LOD) of 1.85 ng/mL and 2.05 ng/mL, respectively, and conducted field tests in rivers.

#### 3.3.3. PFAS

PFAS are a group of synthetic organofluorine compounds. Due to their excellent waterproof, oil-proof, and anti-pollution properties, PFAS are widely used in industrial processing and commercial product manufacturing [209]. PFASs are often referred to as “forever chemicals”, with half-lives up to eight years due to the carbon-fluorine bond, which is one of the strongest bonds in organic chemistry. PFAS have been detected in rain and drinking water, and they are highly mobile and readily absorbed through human skin or ingested through the food chain, causing bioaccumulation. The total costs associated with PFAS-attributed disease are estimated to be around USD 6–62 billion in the United States. More stringent monitoring and regulation are urgently needed to control the health risks posed by PFAS.

Serum protein is the main binding site for PFAS. It has been reported that the binding rates of PFOA and perfluorononanoic acid (PFNA) to human serum albumin (HSA) are 90.0% and 99.9%, respectively [210]. Previous studies have shown that PFOA can bind to, for example, liver fatty acid-binding protein (L-FABP) in serum albumin [211,212,213]. Based on the powerful combination of the two, Mann and Berger designed a fluorescent biosensor using human liver fatty acid-binding protein (hLFABP) for the detection of perfluorooctanoic acid (PFOA). The biosensor combines hLFABP as a recognition unit with a circularly arranged green fluorescent protein (cp.GFP) as a light signal. When PFOA is present in the sample, the binding of PFOA to the cleaved hL-FABP will cause changes in the fluorescence intensity of cp.GFP, which can be used to estimate the PFOA concentration. When the biosensor detects PFOA in environmental water samples, the detection limit is 330 ppb [180]. In addition to the enzyme-based sensors, Park et al. successfully developed an ssDNA aptasensor for the detection of PFAS for the first time. This ssDNA specifically binds to the PFOA with a dissociation constant (KD) of 5.5 μM. A fluorophore is attached to this aptamer and quenched with a cDNA molecule. After the PFOA binds to the aptasensor, the cDNA quench is removed, causing the entire structure to yield fluorescence signals with a detection limit of 0.17 μM. Further research on the binding site of the aptamer and PFOA found that the functional group is not the key factor for the specific binding of the aptamer; rather, the length of the fluorocarbon chain is the most important factor. In addition, bacterial cell-based biosensors have also been successfully developed to detect the PFOA and PFOS in water samples. Pseudomonas aeruginosa (PAO1) and Burkholderia FA1, which have strong biodegradation capability of PFOA and PFOS, were isolated through the enrichment experiments. The corresponding defluorinase enzyme genes were further cloned into the *E. coli* (pRSET-pfc-DEF/gfp) strain. This strain can use the PFOA and PFOS as the substrate to grow and yield detectable fluorescence signals within 24 h. The detection limits for PFOA and PFOS are 10 ng/L to 10 ppm, respectively [182]. 

### 3.4. Comparative Analysis of Biosensor Types

With the increasing demand for the timely and precise detection of ECs in environmental matrices, the development and deployment of biosensors have gained significant attention. However, the diversity of biosensor designs may pose challenges in selecting the most appropriate platform for a given application, driven largely by the choice of biological recognition elements. Different biosensors exhibit distinct characteristics, each offering unique advantages and trade-offs when applied to environmental pollutant detection. Understanding these differences is crucial for selecting the most suitable biosensor design for specific monitoring scenarios, especially when balancing factors such as sensitivity, selectivity, stability, cost, and broader functionality such as toxicity assessment (Table 4).

Enzyme-based biosensors leverage the inherent catalytic activity of enzymes, providing high specificity and sensitivity toward their target substrates. However, they are highly susceptible to environmental stressors such as pH changes, temperature fluctuations, and matrix effects, which can lead to enzyme denaturation and signal loss. Developing or engineering enzymes to detect a broader range of ECs remains technically challenging and costly, further limiting their field applicability.

Immunosensors, built on the strong binding affinity of antibodies, offer versatile detection for a wide variety of contaminants, including small molecules such as antibiotics and pesticides. Despite their high sensitivity and selectivity, the production of antibodies and sensor construction can be expensive and labor-intensive. Moreover, the irreversible nature of antigen–antibody binding typically prevents sensor regeneration, reducing reusability.

Aptasensors, derived from chemically synthesized oligonucleotides, present significant advantages in the stability, scalability, and ease of functional modification. Nonetheless, the SELEX process used to develop aptamers is time-consuming, and the resulting aptamers often exhibit lower binding affinity than antibodies, compromising sensitivity in some applications. In addition, aptamers can be degraded by nucleases in environmental samples or lose their structure in complex matrices, which impacts long-term performance.

Cell-based biosensors are relatively robust due to the natural self-protection mechanisms of living cells and can integrate complex responses to environmental toxicity. They can operate in diverse and untreated environmental samples, making them particularly suitable for on-site toxicity assessment. However, they generally exhibit slower response times and lower quantitative precision due to multi-step metabolic pathways and biological variability. While cell-free expression systems have been proposed to improve stability and reduce variability, they remain in the early stages of field validation.

## 4. Challenges of the Application of Biosensors in Monitoring Emerging Contaminants in the Water Environment

Building upon the comparative performance analysis in Section 3.4, this section outlines the key challenges and limitations that currently hinder the field deployment and real-world application of biosensors for environmental monitoring. Because of ECs’ widespread occurrence and potential health risks, stricter regulations, timely monitoring, and effective remediation are essential to support One Health practices and ensure the sustainability of both human and environmental ecosystems. Biosensors provide a promising alternative to conventional monitoring methods by offering rapid, cost-effective (once optimized), and portable detection systems. They also hold potential for integration into on-site devices and household applications, potentially democratizing access to water quality information [106]. However, despite these advantages, biosensors still face several challenges related to sustainability, reusability, and field applicability, which have hindered their widespread implementation.

Despite the advantages, biosensors still face several key challenges, particularly regarding their translation from laboratory research to field-ready applications. Many biosensors have been validated under controlled laboratory conditions but have not yet been sufficiently tested in real-world environmental matrices. In actual water samples, matrix effects can significantly interfere with sensor accuracy and stability, such as the presence of humic substances, suspended solids, heavy metals, or variable pH [163]. These conditions often necessitate additional sample pretreatment steps, such as filtration, dilution, or pH adjustment, which limit the feasibility of on-site deployment. To perform reliably in such complex environments, biosensors must possess high specificity, environmental tolerance, and minimal signal drift.

Furthermore, as research on environmental pollutants expands, new types of ECs are continuously being identified. This demands biosensor platforms capable of multiplexed detection to monitor multiple targets simultaneously, placing greater emphasis on signal amplification strategies and system scalability. Standardization also remains a bottleneck. The diversity of biosensor types makes it difficult to establish unified protocols for calibration, validation, and performance comparison, due to each type employing different recognition elements, binding mechanisms, and signal transduction pathways.

In addition, technical parameters such as sensitivity, operational stability, cost-efficiency, and reusability still require improvement. These limitations constrain their long-term field use and hinder large-scale adoption. Finally, regulatory approval pathways for biosensors in environmental monitoring are still evolving, and the lack of clear quality control and certification frameworks continues to delay real-world implementation [214]. Addressing these challenges is essential to unlocking the full potential of biosensors for routine and widespread environmental surveillance.

Enzyme-based biosensors exhibit high specificity by leveraging natural catalytic activity but suffer from poor adaptability. Finding or engineering enzymes for diverse ECs remains costly and technically demanding, with uncertain outcomes. Their performance is also sensitive to environmental stressors such as pH shifts, temperature fluctuations, and ionic strength, often leading to denaturation or signal loss in complex samples.

Immunosensors offer better flexibility, as antibodies can be customized to detect a wide variety of contaminants, including small-molecule haptens. Yet, their development involves high production costs, particularly in antibody generation and signal amplification mechanisms, which often require additional labeling steps. In addition, the irreversible nature of antigen–antibody binding generally limits sensor reusability, as the recognition elements cannot be easily regenerated.

Aptasensors benefit from chemical synthesis and ease of functional modification, making them attractive for field applications. However, the SELEX process is laborious and time-consuming, and the resulting aptamers often show lower affinity than antibodies or enzymes, compromising sensitivity. Moreover, aptamer degradation and conformational instability in complex matrices limit long-term usability.

Cell-based biosensors are robust and adaptable due to natural cell protection and genetic engineering tools. They can function in diverse environmental matrices and offer low-cost platforms. Nevertheless, they often suffer from long response times and less precise quantification due to multi-step metabolic pathways and inherent biological variability. Cell-free expression systems offer some improvements in stability and control but are still in the early stages of field validation.

## 5. Future Directions for Biosensor Development for Emerging Contaminants Detection

Immobilization on solid surfaces is a widely used strategy to enhance the stability, sensitivity, and functionality of biosensors. Polymers play a critical role in constructing these solid surfaces and are generally categorized into conductive and non-conductive types. Conductive polymers encapsulate the electrode surface to facilitate signal transmission, while non-conductive polymers are used to immobilize specific receptors on the sensor platform [215]. Nanomaterials provide an alternative and often synergistic approach to improve biosensor performance in ECs detection. Commonly used nanomaterials include metal and metal oxide nanoparticles, such as AuNPs, AgNPs, and iron oxide NPs [216]. Among these, MNPs have attracted particular interest for their potential in sensor recovery using magnetic fields, especially for aptasensors, thereby significantly reducing the cost of biosensor analysis.

In addition to focusing on the biological receptor part, other modules in the biosensor also have a certain impact on its performance. Compared with common transducers, field effect transistor (FET)-based biosensors have the advantages of high sensitivity, fast response speed, real-time signal amplification, easy miniaturization, and integration for high-throughput screening [217]. Cristian et al. further optimized the high-performance glucose biosensor by integrating glucose oxidase (GOx) with MOS transistors on silicon wafers. Their study deposited a 90 nm titanium (Ti) film in the gate area and converted it into nanostructured TiO_2_ to improve the uniformity and adhesion of the enzyme film, thereby further improving the detection range and sensitivity [218]. With the progress in various fields, such as materials science, synthetic biology, and genetic engineering, research breakthroughs in various modules of biosensors have also promoted the improvement of sensor performance and applicability.

To further enhance analytical characteristics, hybrid nanostructures combining nanomaterials with polymers or other nanomaterials have gained attention. These composites can yield synergistic effects, significantly improving sensitivity, extending the linear detection range, reducing testing time, and enhancing long-term operational stability [219,220,221].

The integration of microfluidics and machine learning has also revolutionized biosensor development. Microfluidic platforms are ideal for on-site ECs detection, offering precise sample control, enhanced portability, and reduced reagent consumption. Machine learning algorithms can analyze complex sensor outputs, improving both sensitivity and specificity. For instance, a microfluidic biosensor combined with laser printing enabled real-time detection of multiple antibiotics with improved accuracy and reduced false positives [222]. Similarly, a fluorescence sensor array based on diverse copper nanoclusters (Cu NCs), when integrated with machine learning, accurately identified multiple heavy metals and pesticides within 10 min, achieving detection limits as low as 7.1 ppb [223]. These integrated systems offer the potential for intelligent, automated platforms capable of the simultaneous monitoring of multiple pollutants with high precision.

The development of portable and user-friendly biosensors is another promising direction, including cartridge-based devices, miniaturized microfluidic systems, and paper-based formats [132]. This trend is driven by the rising awareness of water quality and the demand for rapid, field-deployable solutions. ECs pose significant risks to both human health and ecological systems, such as pharmaceuticals, antibiotics, pesticides, and microplastics. Real-time, source-level detection in households, industries, or remote areas is essential for timely mitigation. A notable example is a smartphone-based whole-cell biosensor device for salicylic acid detection, featuring gelatin-based hydrogel-encapsulated sensors on a glass slide and a linear detection range of 0.1 to 10 µM. The accompanying smartphone app enables on-site fluorescence-based analysis [224].

Additionally, future biosensors may enable multifunctional monitoring, allowing for the simultaneous detection of multiple parameters in a single device. Integration with wastewater epidemiology, for example, could facilitate not only EC monitoring but also the detection of pathogens, viruses, and community-level health indicators, such as pharmaceutical usage [225,226]. This advancement could transform public health surveillance by enabling early outbreak detection and pollution source tracking. Furthermore, these multifunctional biosensors could empower individuals to monitor drinking water and household wastewater conveniently, affordably, and in real time.

While these technological innovations are promising, broader systemic and regulatory challenges must also be addressed to ensure successful real-world implementation. Moving forward, focused efforts are needed to establish standardized protocols for calibration, validation, and performance benchmarking across various biosensor platforms. These protocols should consider the diversity of environmental matrices, potential interference from background compounds, and consistency in analytical performance to support regulatory recognition. Equally important is advancing the design of multiplexed biosensors capable of simultaneously detecting multiple ECs in complex sample conditions. This will require integrating signal amplification techniques, microfluidic control, and real-time data processing through computational tools such as machine learning. In parallel, addressing regulatory and commercialization hurdles is essential. The absence of a harmonized framework for biosensor certification in environmental applications remains a significant barrier to market deployment. Strengthening collaboration among researchers, industry stakeholders, and regulatory agencies will be vital to developing clear evaluation criteria and facilitating the adoption of biosensors in environmental monitoring systems.

## 6. Conclusions

ECs have become one of the grand challenges threatening water safety, human health, and environmental health in the 21st century. Biosensors are emerging as a promising approach for the fast, economic, and on-site monitoring of various types of ECs in water environments, supporting scientific health risk assessment and evidence-based regulatory decisions. In this review, we provide a comprehensive overview of biosensor types, their working mechanisms, recent advancements in detecting ECs, and current challenges, particularly detection capability and stability. One promising optimization strategy is the immobilization of biosensors on hybrid nanomaterials and polymers. Another key area of focus is the development and engineering of novel biosensor devices that combine integrated microfluidics, machine learning, and sensor array technologies with biosensors to achieve more sensitive, portable, multifunctional, and user-friendly detection of ECs. Therefore, environmental scientists are encouraged to collaborate with researchers from fields outside of environmental science and engineering, such as microbiology, analytical chemistry, molecular biology, and biological engineering. Such interdisciplinary collaboration would drive revolutionary innovations in biosensor technology, improving our quality of life and helping us address the grand challenges we currently face. With their broad application potential, we anticipate that biosensors could become a highly effective tool for monitoring various contaminants across diverse scenarios.

## Figures and Tables

**Table 1:** Summary of various types of biosensors for pesticide detection.

Biosensing Element	ECs		Transducer	Detection Limit	Detection Range	Response Time	References
Enzyme-based biosensors							
Phosphorylated acetylcholinesterase (AChE)	Organophosphorus pesticides		Electrochemical	0.05–2.03 nM	2.85–1 × 10^4^ nM	20 min	[54]
	Dichlorovos		/	0.0087–0.029 nM	0.0716–2.08 nM	/	[55]
	Chlorpyrifos		/	0.0014–0.0046 nM	0.0358–0.711 nM	/	
	Paraxon		Amperometric	7.27 nM	up to 14.54 nM	/	[56]
	Parathion		Electrochemical	4.9 × 10^−13^ M	10^−12^–10^−6^ M	10 min	[57]
	Dichlorvos		Amperometric	13.734–31.676 nM	16–28 nM	4 min	[58]
	Monocrotophs						
	Parathion						
	Chlorpyrifos		Amperometric	0.057 nM	0.1426–285.17 nM	15 min	[59]
Butyrylcholinesterase (BChE)	Paraoxon		Electrochemical	18 nM	Up to 100 nM	/	[60]
Kidney bean esterase (KdBE)	Chlorpyrifos		Electrochemical	9.98 nM	28.52–1.4 × 10^6^ nM	15 min	[61]
Dehydrohalogenase	1,1,1-trichloro-2,2-bis (ρ-chlorophenyl) ethane (DDT), Hexachlorocyclohexane (HCH)		Electrochemical	/	0.01–12 mM	10 to 15 min	[62]
Antibody/antigen-based biosensors (Immunosensors)							
Anti-chlorpyrifos	Chlorpyrifos		Field effect transistors	1.8 fM	1 fM–1 μM	/	[63]
Anti-imidacloprid	Imidacloprid		Chronoamperometric	22 pM	50–10,000 pM	60 s	[64]
Anti-triazophos	Triazophos		Fluorescence	0.0223 nM	0.032–63.84 nM	150 min	[65]
Anti-parathion	Parathion		Fluorescence	0.031 nM	0.1717–171.6752 nM	150 min	
Anti-chlorpyrifos	Chlorpyrifos		Fluorescence	0.2481 nM	1.4264–2851.7 nM	150 min	
Anti-carbofuran (monoclonal antibody) and Anti-3-hydroxycarbofuran (monoclonal antibody)	Carbofuran and 3Hydroxycarbofuran		Lateral flow immunochromatography assay	29.5–45.2 nM	0–90.4 nM	5 min	[66]
Nucleic acid-based biosensors (Aptasensors)							
Three Single-stranded DNAs (ssDNA)	Acetamiprid		Fluorescence	5.73 nM	0–500 nM	/	[67]
Complementary Strands 1/Complementary Strands 2-FAM/Complementary Strands 3-AuNPs/Aptamer	Acetamiprid	Fluorescence		2.8 nM	5 50 nM	/	[68]
Fourier transform infrared (FT-IR) spectra: Cationic carbon dots (cCDs)	Acetamiprid	Fluorescence		0.3 nM	1.6–20 nM	30 min	[69]
Rhodamine B/Aggregation of AuNPs	Carbendazim	Fluorescence		2.33 nM	2.33–800 nM	/	[70]
Whole cell-based biosensors							
An *Escherichia coli* strain carrying a chpR expression vector and a chpA promoter eatsBA transcriptional fusion plasmid encoding sulfatase (atsA) and formylglycine generating enzyme (atsB) from *Klebsiella* sp.	Chlorpyrifos	Fluorescence		/	25–500 nM	/	[71]
*Escherihia coli*	Paraoxon	Electrochemical		9 nM	0.05–25 μM	/	[72]
	Parathion			10 nM	0.05–25 μM	/	
	Methylparathion			15 nM	0.08–30 μM	/	
	Lindane	Electrochemical		/	0.0069–0.1547 nM t	/	[73]
*Streptomyces* strain M7	Lindane	Electrochemical		412.61 nM	/	2 days	[74]
*Escherihia coli* SM003	Atrazine	Fluorescence		1.08 μM	1.08–15 μM	/	[75]
*Escherihia coli* SM003	Cyanuric Acid	Fluorescence		7.83 μM	7.83 μM–2.89 mM	/	
*Escherihia coli* SM004	Cyanuric Acid	Fluorescence		0.22 μM	0.22–15 μM	/	

**Table 2:** Summary of various types of biosensors for antibiotic detection.

Biosensing Element	ECs	Transducer	Detection Limit	Detection Range		Response Time	References
Enzyme-based biosensors							
Laccase	Paracetamol	Optical	0.55 × 10^−6^ M	2–14 μM		20 s	[114]
Penicillinase	penicillin G	Amperometric	4.5 nM	10–50 nM		/	[115]
Tyrosinase	Methimazole	Amperometric electrochemical	0.004–0.006 μM	0.01–10 μM		20 s	[116]
	Captopril		0.008–0.019 μM	0.05–15 μM		/	[117]
Monoamine oxidase	Imipramine	Amperometric	7 × 10^−9^ M	1 × 10^−8^–5 × 10^−5^ M		/	[118]
	Amitriptyline		8 × 10^−9^ M				
Antibody/antigen-based biosensors (Immunosensors)							
Anti-sulfamethazine monoclonal antibody	Sulfamethazine	Electrochemical	0.43 nM	1.19–229.25 nM		30 min	[119]
Anti-penicillin monoclonal antibody (antiP)	Penicillin G	Amperometric	0.2 nM	0.5–5.98 × 10^4^ nM		45–55 min	[120]
Anti-ampicillin, monoclonal antibody	Ampicillin	Direct-flow surface plasmon resonance	0.25 nM	0.5–9.988 × 10^−2^ nM		15 min	[121]
Anti-chloramphenicol, monoclonal antibody	Chloramphenicol	Amperometric	0.0145 nM	0.03–31 nM		30 min	[122]
Anti-tetracycline monoclonal antibody	Tetracycline	Electrochemical	0.18–2.25 nM	0.0722 nM		20 min	[123]
Anti-tetracycline	Tetracycline	Electrochemical	10^−6^–10^−14^ M	3.8 × 10^−15^ M		/	[124]
Anti-kanamycin	Kanamycin	Electrochemical	0.1–33 nM	0.03 nM		/	[125]
Nucleic acid-based biosensors (Aptasensors)							
Non-cross-linking deaggregation of AuNPs coated with a polyadenine-modified aptamer	Chloramphenicol	Colorimetric	22 nM	0.19–3.19 nM		30 min	[126]
Quenching of FAM-labeled aptamer by target-responsive GO hydrogel	Oxytetracycline	Fluorescence	5.43 × 10^4^ nM	5.43 × 10^4^–2.17 × 10^6^ nM		/	[127]
Utilization of AuNPs modified magnetic beads and fi nicking enzyme	Ampicillin	Fluorescence	0.2 nM	0.29–286 nM		/	[128]
Salt-induced aggregation of AuNPs in the presence of target	Oxytetracycline	Colorimetric	10 nM	25–2500 nM		/	[129]
Thiols modified aptamer and methacrylic acid	Kanamycin	Fluorescence	26.8 nM	103–2 × 10^4^ nM		25 min	[130]
AuNPs functionalized with the hemin/Gquadruplex DNAzyme and cDNA	Chloramphenicol	Colorimetric	402 nM		3.1 × 10^−3^–309 nM	60 min	[131]
Whole cell-based biosensors							
*Escherichia coli*	Tetracycline	Filter paper strips	11.76–38.47 nM		168–2.2 × 10^4^ nM	90 min	[132]
			11.7–79.4 nM		168–1.6 × 10^5^ nM		
	Chlortetracycline	Fluorescence	6.6 nM		/	60 min	[133]
	Oxytetracycline		9.33 nM				
	Doxycycline		7.154 nM				
	Minocycline		13.6 nM				
	Metacycline		10 nM				
	Demeclocycline		13.3 nM				
	Tigecycline		39 nM				
	Tetracycline		71 nM				
*S. oneidensis*	Ampicillin	Fluorescence	73 nM		/	/	[134]

**Table 3:** Summary of various types of biosensors for endocrine disruptors (EDCs), persistent organic pollutants (POPs), and per-and polyfluoroalkyl substances (PFAS) detection.

ECs	Biosensing Element	Detected	Transducer	Detection Limit	Detection Range	References
EDCs	Antibody/antigen-based biosensors (Immunosensors)					
	Monoclonal antibodies of 17β-Estradiol	17β-estradiol	Electrochemical	0.4 × 10^−3^ nM	0.4 × 10^−3^–0.73 nM	[171]
	PDA NS/Mn:ZnCdS-anti-E2	17β-estradiol	Photoelectrochemical	1.1 × 10^−3^ nM	1.8 × 10^−3^–73 nM	[172]
	Anti-DES	Diethylstilbestrol	Photoelectrochemical	0.2 × 10^−3^ nM	0.4 × 10^−3^–74 nM	[173]
	Nucleic acid-based biosensors (Aptasensors)					
	Raman reporter molecule Cy3 labeled E2-aptamer	17β-estradiol	Surface-enhanced Raman scattering (SERS)	2.75 fM	1.0 × 10^−13^–1.0 × 10^−9^ M	[174]
	Aptamer against BPA	Bisphenol A	Fluorometric	3.3 nM	10–900 nM	[175]
	Aptamer against BPA	Bisphenol A	Electrochemiluminescence	30 fM	0.1 p1 nM	[176]
	Shortening DNA aptamer	17β-estradiol	Colorimetric	0.1 nM	0.2–5 nM	[177]
PFAS	Antibody/antigen-based biosensors (Immunosensors)					
	Mono-specific antibody against the PFOA	PFOA and PFOS	Optical fiber	0.604 nM	/	[178]
	Nucleic acid-based biosensors (Aptasensors)					
	ssDNA aptamers	PFOA	Fluorometric	0.17 μM	>5 μM	[179]
	Whole cell-based biosensors					
	Conjugating circularly permuted green fluorescent protein (cp.GFP) to a split-hLFABP construct	PFOA	Fluorometric	330 ppb	/	[180]
	Genetically engineered bacteria	PFOA and PFOS	Fluorometric	0.314 nM	0.02–2 nM	[181]
	*P. aeruginosa* (PAO1)	PFOA and PFOS	Fluorometric	/	0.02–24.14 nM	[182]
POPs	Enzyme-based biosensors					
	Horseradish peroxidase	Polybrominated diphenyl ethers-100	Amperometric	24.8 pM	751–45745 pM	[183]
		Polybrominated biphenyls-1		57.5 pM	2.75–42.8 nM	
		Polychlorinated biphenyls-1		75.3 pM	3.19–61.9 nM	
		Polychlorinated biphenyls-28		48.9 pM	2.23–48 nM	
		Polychlorinated biphenyls-101		58.3 pM	2.85–83.1 nM	
	Antibody/antigen-based biosensors (Immunosensors)					
	Olyclonal anti-polychlorinated biphenyl (PCB) antibody	Polychlorinated biphenyls-28	Electrochemical	193 nM	612–3674 nM	[184]
	Nucleic acid-based biosensors (Aptasensors)					
	BHQ-Cy5 modified single-stranded DNA	3,3′,4,4′-tetrachlorobiphenyl	Fluorometric	0.438 fM	1.71 fM–171 μM	[185]
	ssDNA aptamers	PCB-77	Surface-enhanced Raman spectroscopy (SERS)	3.3 × 10^−8^ M	3.3 × 10^−8^–1.0 × 10^−7^ M	[186]
	Aptamer	Polychlorinated biphenyls-77	Colorimetric	0.05 nM	0.5–900 nM	[187]
	Whole cell-based biosensors					
	Two harpins (H1 and H2) were designed according to the partial complementary sequence (cDNA) of the PCB72/106	PCB72 and PCB106	Fluorescent	8.86 pM and 9.68 pM	10.1 pM–2.02 μM and 11.1 pM–2.21 μM	[188]

**Table 4:** Comparative performance of enzyme-, antibody-, aptamer-, and cell-based biosensors for the environmental monitoring of emerging contaminants.

Biological Receptors	Sensitivity	Selectivity	Stability	Cost	Toxicity Assessment	Broad-Spectrum Analysis
Enzymes	High	High	Low-Moderate	Low-Moderate	Poor	Moderate
Antibodies	Very High	Very High	Moderate	High	Poor	Poor
Aptamers	High	Very High	Very High	Low-Moderate	Poor	Poor
Cells	Moderate	Moderate	Very High	High	Very High	Very High
